# Up-regulation of C1GALT1 promotes breast cancer cell growth through MUC1-C signaling pathway

**DOI:** 10.18632/oncotarget.3045

**Published:** 2015-01-27

**Authors:** Chih-Hsing Chou, Miao-Juei Huang, Chi-Hau Chen, Ming-Kwang Shyu, John Huang, Ji-Shiang Hung, Chiun-Sheng Huang, Min-Chuan Huang

**Affiliations:** ^1^ Graduate Institute of Anatomy and Cell Biology, National Taiwan University College of Medicine, Taipei, Taiwan; ^2^ Department of Obstetrics and Gynecology, National Taiwan University Hospital, Taipei, Taiwan; ^3^ Department of Surgery, National Taiwan University Hospital, Taipei, Taiwan; ^4^ Research Center for Developmental Biology and Regenerative Medicine, National Taiwan University, Taipei, Taiwan

**Keywords:** Breast cancer, C1GALT1, T antigen, MUC1, beta-catenin

## Abstract

Aberrant glycosylation is frequently observed in cancers. Core 1 β1,3-galactosyltransferase (C1GALT1) is an exclusive enzyme in humans that catalyzes the biosynthesis of core 1 O-glycan structure, Gal-GalNAc-O-Ser/Thr, whose expression is commonly up-regulated during tumorigenesis. Little is known about the function of C1GALT1 in breast cancer. This study aims to determine the correlation between C1GALT1 expression and breast cancer clinicopathological features and roles of C1GALT1 in breast cancer malignant phenotypes. Public databases and our data showed that *C1GALT1* mRNA and C1GALT1 protein are frequently up-regulated in breast cancer; and increased C1GALT1 expression correlates with higher histological grade and advanced tumor stage. Overexpression of C1GALT1 enhanced breast cancer cell growth, migration, and invasion *in vitro* as well as tumor growth *in vivo*. Conversely, C1GALT1 knockdown suppressed these malignant phenotypes. Furthermore, C1GALT1 modulates O-glycan structures on Mucin (MUC) 1 and promotes MUC1-C/β-catenin signaling in breast cancer cells. These findings suggest that C1GALT1 enhances breast cancer malignant progression through promoting MUC1-C/β-catenin signaling pathway. Unveiling the function of C1GALT1 in breast cancer opens new insights to the roles of C1GALT1 and O-glycosylation in tumorigenesis and renders the potential of C1GALT1 as a target of novel therapeutic agent development.

## INTRODUCTION

Breast cancer is the most diagnosed malignancy in women with the highest cancer incidence rate reported worldwide. Approximately 10–15% of patients with breast cancer has an aggressive disease and develops distant metastases within 3 years after the initial detection of primary tumor [1, 2]. However, effective therapies to reduce metastasis or recurrence remain a critical challenge.

Glycosylation is the most common post-translational modification in mammalian cells, where N-linked and O-linked glycosylation are most frequent occurring. Mucin-type O-glycosylation is the most common O-linked glycosylation [3, 4]. During mucin-type O-glycosylation, the N-acetylgalactosaminyltransferase (GALNT) family enzymes initiate the biosynthesis of GalNAc-O-Ser/Thr structure, also known as Tn antigen, by transferring N-acetylgalactosamine (GalNAc) from UDP-GalNAc to serine (Ser) or threonine (Thr) residues. Subsequently, core 1 β1,3-galactosyltransferase (C1GALT1), an exclusive T-synthase in mammalian cells, catalyzes the transfer of galactose (Gal) from UDP-Gal to form Gal-GalNAc-O-Ser/Thr structure, known as T antigen, as basis for further complex O-glycan formation, such as core 2 structure.

Aberrant O-glycosylation is commonly found in many cancers, including breast cancer, involving in the pathophysiological processes of tumor development, including tumor cell proliferation, migration, invasion, metastasis and angiogenesis [5, 6]. GALNT6 affects mammary tumorigenesis by altering glycosylation of MUC1 oncoprotein [7] and fibronectin [8]. Relocation of GALNT2 from Golgi to endoplasmic reticulum enhances Tn antigen expression in breast cancer [9]. ST3GAL1 and ST6GALNAC1, producing sialylated T and Tn antigens, respectively, have been found to involve in mammary tumorigenesis [10, 11].

ClGALT1 plays critical roles in many biological functions; and its altered expression results in developmental defects and affects cancer malignant behaviors. Knockout of C1GALT1 causes spontaneous colitis [12] and thrombocytopenia [13] in mice and modulates platelet cell surface glycoproteins involved in platelet synthesis and hematopoiesis [14]. We recently reported that C1GALT1 alters O-glycan structures on MET and enhances MET dimerization in hepatocellular carcinoma [15]; and C1GALT1 modifies O-glycans on FGFR2 and its downstream signaling involved in colorectal cancer malignant phenotypes [16]. Several studies reported that T antigens are overexpressed in breast cancer metastatic tumor cells and overexpression of T antigen regulates breast cancer malignant phenotypes [6, 17–19]. High expression levels of T antigens were found on MUC1 [20–22] and promoted breast cancer metastasis through galectin-3 [23]. Although C1GALT1 plays critical roles in many pathophysiological functions, the expression and function of C1GALT1 in breast cancer are still unclear. Here, we report that C1GALT1 is overexpressed in breast cancer and its overexpression enhances malignant growth of breast cancer cells through modifying MUC1 O-glycosylation and signaling. We also demonstrate that inhibiting C1GALT1 is sufficient to suppress tumor growth *in vitro* and *in vivo*, suggesting that C1GALT1 may serve as a therapeutic target for breast cancer treatment.

## RESULTS

### *C1GALT1* mRNA and C1GALT1 protein are up-regulated in breast cancer

**Figure 1:**
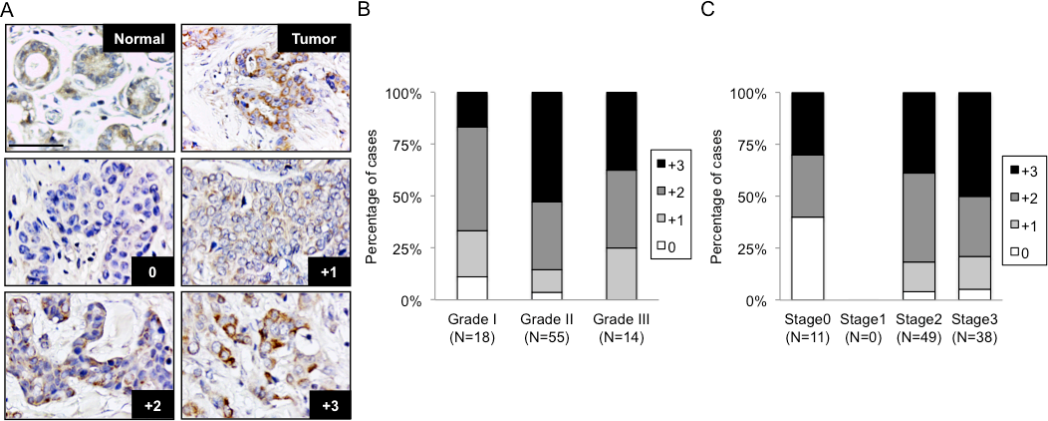
C1GALT1 is frequently overexpressed in breast tumor tissues and correlates with histological grade and stage **(A)** Immunohistochemical staining of C1GALT1 in normal mammary tissues and breast tumor tissues. Scale bars = 50 μm. Negative control did not show any specific signals (data not shown). Staining intensity of C1GALT1 was scored from 0, +1, +2, +3 and is graphed against breast cancer histological grade **(B)** and tumor stage **(C)**. N indicates patient number.

Data retrieved from public databases show that *C1GALT1* mRNA expression levels are up-regulated in ductal breast carcinoma (*n* = 40) compared with normal (*n* = 7) (Oncomine) (Supplementary Figure S1A). *C1GALT1* expression levels are also up-regulated across a selection of 9 breast cancer biosets (NextBio Research) (Supplementary Figure S1B). Consistently, immunohistochemical staining of C1GALT1 in a breast cancer tissue microarray shows that C1GALT1 protein is frequently overexpressed in breast cancer tissues compared with normal tissues (Figure 1A). These results indicate that *C1GALT1* mRNA and C1GALT1 protein are commonly up-regulated in breast cancer tissues compared with normal breast tissues.

### Higher C1GALT1 expression correlates with higher breast cancer histological grade and advanced tumor stage

The staining intensity of C1GALT1 was scored according to the percentage of C1GALT1-positive cells in each tissue (0, negative; +1, <20%; +2, 20–50%; +3, >50%) (Figure 1A). C1GALT1 intensity is plotted against histological grade (Figure 1B) and tumor stage (Figure 1C). Score 0 and +1 were considered as low expression; and +2 and +3 were considered as high expression. Chi-square analysis shows that high C1GALT1 expression correlates with advanced tumor stage (Table 1). Spearman Rank Correlation analysis reveals that high C1GALT1 expression correlates with higher histological grade and advanced tumor stage. These results suggest a role of C1GALT1 in breast cancer development.

**Table 1:** C1GALT1 expression level correlates with clinicopathological characteristics

	C1GALT1 expression				
Factor	0	+1	+2	+3	*p* value
Grade I	2	4	9	3	**p < 0.01
Grade II	2	6	18	29	Spearman Rank Correlation
Grade III	0	4	6	6	
**Factor**	**0**	**+1**	**+2**	**+3**	
Stage 0	4	4	3	0	**p < 0.01
Stage 1	0	0	0	0	Spearman Rank Correlation
Stage 2	2	7	21	19	
Stage 3	2	6	11	19	
**Factor**	**Low (N = 25)**	**High (N = 73)**			
Stage 0	8	3			**p < 0.01
Stage 1	0	0			Chi-square
Stage 2	9	40			
Stage 3	8	30			

* *p* < 0.05

** *p* < 0.01

### C1GALT1 regulates O-glycan structures on surfaces of breast cancer cells

**Figure 2:**
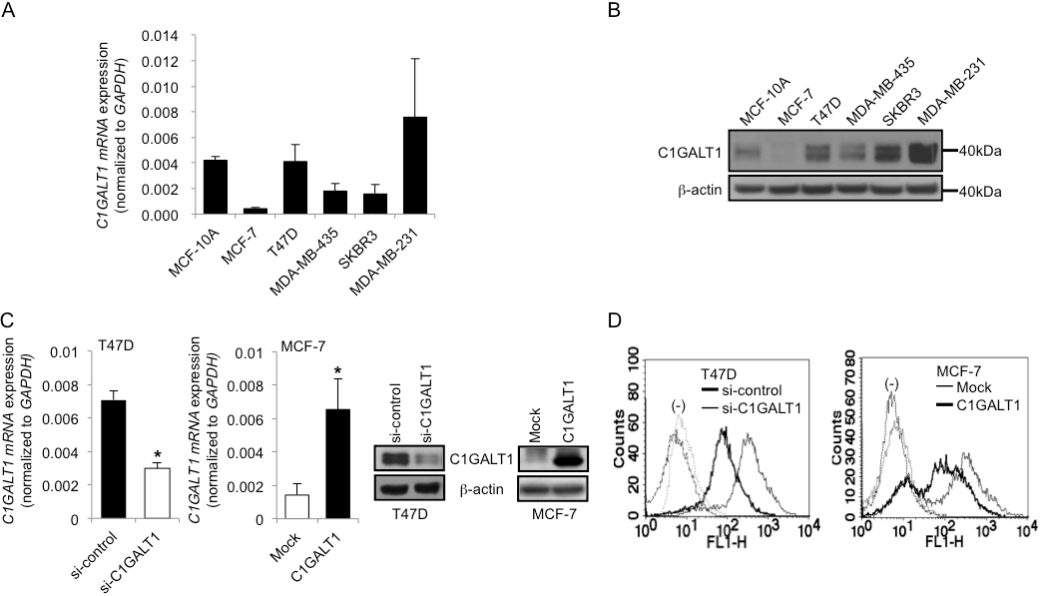
C1GALT1 regulates O-glycan structures on surfaces of breast cancer cells *C1GALT1* mRNA **(A)** and C1GALT1 protein **(B)** expression levels in breast cancer cell lines analyzed by Q-RT-PCR and Western blotting, respectively. **(C)** Knockdown and overexpression of C1GALT1 in T47D and MCF-7 cells confirmed by Q-RT-PCR and Western blotting, respectively. T47D cells were transfected with non-target (si-control) or C1GALT1 specific (si-C1GALT1) siRNA. MCF-7 cells were transfected with empty vector (Mock) or C1GALT1/pcDNA3.1 plasmid (C1GALT1). **(D)** Effects of C1GALT1 expression on cell surface O-glycans analyzed by flow cytometry. VVA lectin conjugated with FITC was applied to analyze cell surface Tn antigen expression in T47D and MCF-7 transfectants.

C1GALT1 mRNA and protein expression levels in breast cancer cell lines, including MCF-10A, MCF-7, T47D, MDA-MB-435, SKBR3, and MDA-MB-231, were analyzed by Q-RT-PCR and Western blotting, respectively. C1GALT1 expression levels are higher in T47D, SKBR3 and MDA-MB-231 cells and lower in MCF-10A and MCF-7 cells (Figure 2A and 2B). C1GALT1 knockdown by specific siRNA in T47D cells and overexpression with pcDNA3.1A/C1GALT1 plasmids in MCF-7 cells were confirmed by Q-RT-PCR and Western blotting (Figure 2C). To investigate whether C1GALT1 expression can modify O-glycan expression on breast cancer cell surfaces, we performed flow cytometry with *Vicia villosa* agglutinin (VVA) lectin, which is specific for GalNAc (Tn antigen) binding. Flow cytometry reveals that knockdown of C1GALT1 enhanced VVA binding to cell surfaces of T47D cells, while overexpression of C1GALT1 decreased VVA binding to MCF-7 cells (Figure 2D). Furthermore, we analyzed T-synthase activity in T47D and MCF-7 transfectants (Supplementary Figure S2). Knockdown of C1GALT1 significantly decreased T-synthase activity in T47D cells (Supplementary Figure S2A) and overexpression of C1GALT1 significantly increased T-synthase activity in MCF-7 cells (Supplementary Figure S2B). These results suggest that the expression of C1GALT1 regulates cell surface O-glycan structures of breast cancer cells.

### C1GALT1 regulates malignant behaviors of breast cancer cells

**Figure 3:**
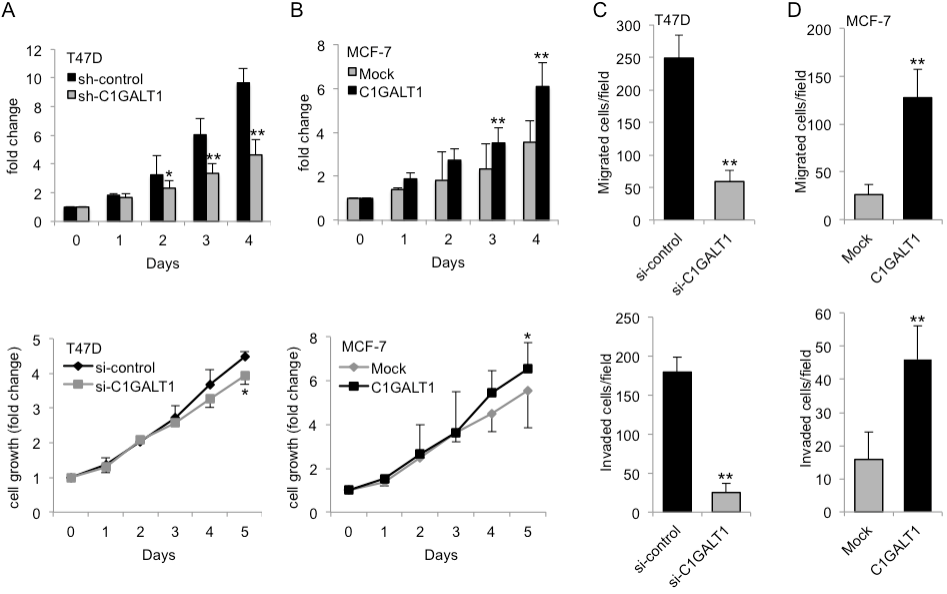
C1GALT1 modulates breast cancer malignant behaviors **(A)** C1GALT1 knockdown in T47D cells suppressed cell viability and cell growth analyzed by MTT and trypan blue exclusion assays. Upper panel, T47D cells stably knocked down with shRNA were seeded into 12-well culture plates and live cells were analyzed by trypan blue exclusion assay and counted with hemocytometer for day 0 to 4. Lower panel, control or C1GALT1 knockdown T47D cells (2 × 10^3^) were seeded into a 96-well plate and incubated for 5 day. The MTT assay results were analyzed by spectrophotometer and normalized to day 0. **(B)** Overexpression of C1GALT1 in MCF-7 cells enhanced cell viability and cell growth analyzed by MTT and trypan blue exclusion assay. Effects of C1GALT1 on cell migration and invasion of T47D cells **(C)** and MCF-7 cells **(D)**. Control or C1GALT1 knockdown T47D cells and mock or C1GALT1 overexpressing MCF-7 cells (5 × 10^4^) in serum free DMEM medium were seeded into the upper chamber of transwell, and 10% FBS in DMEM was added to the lower chamber as a chemoattractant for migration assay. Matrigel™ (BD Biosciences) coated transwells were used in invasion assay. The number of migrated and invaded cells was counted from three fields under microscope. Data are represented as mean ± SD from three independent experiments. *p < 0.05; **p < 0.01.

To investigate the role of C1GALT1 in breast cancer malignant behaviors, cell growth, migration, and invasion were analyzed. MTT and trypan blue exclusion assays show that knockdown of C1GALT1 decreased cell growth in T47D cells (Figure 3A), whereas overexpression of C1GALT1 enhanced cell growth in MCF7 cells (Figure 3B). In addition, Western blotting of a second *C1GALT1* specific siRNA (si-C1GALT1#2) was performed to confirm the effect of C1GALT1 in T47D cells (Supplementary Figure S3A), and the result show that knockdown of C1GALT1 significantly decreased cell growth compared with control (Supplementary Figure S3B). In transwell migration and matrigel invasion assays, C1GALT1 knockdown suppressed T47D cell migration and invasion, while C1GALT1 overexpression promoted MCF-7 cell migration and invasion (Figure 3C and 3D). These studies suggest that C1GALT1 expression enhances cell growth, migration and invasion in breast cancer cells.

### C1GALT1 regulates breast cancer tumor growth *in vivo*

**Figure 4:**
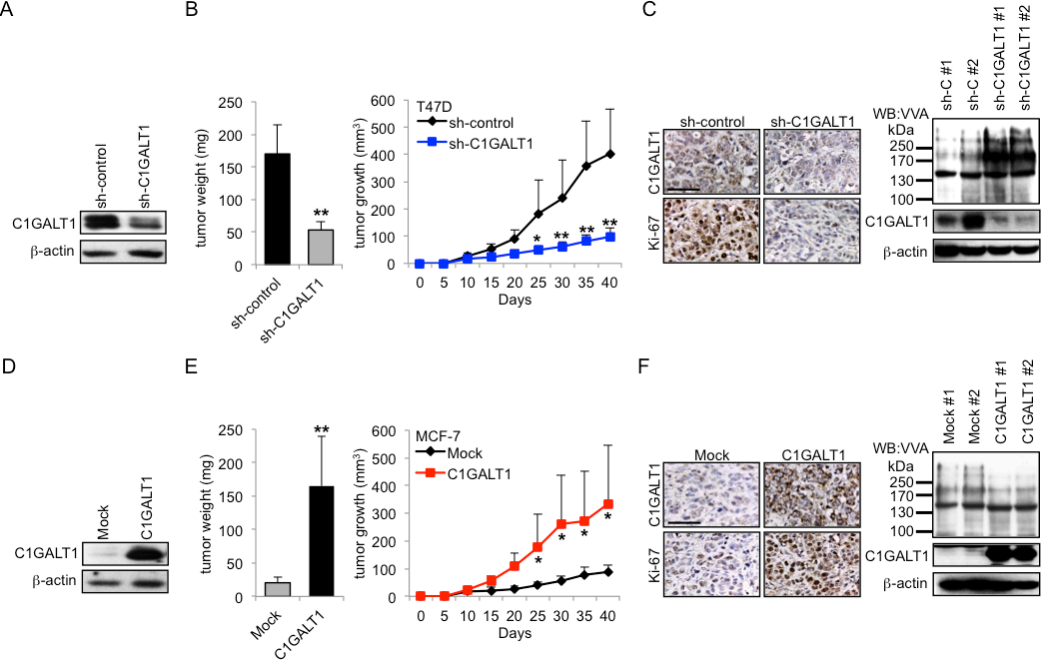
C1GALT1 promotes breast tumor growth *in vivo* **(A)** Stable knockdown of C1GALT1 in T47D cells by C1GALT1 specific shRNA. C1GALT1 protein expression level was analyzed by Western blotting. **(B)** Knockdown of C1GALT1 in T47D cells suppressed tumor weight and tumor growth in NOD/SCID mice. Tumor growth was measured every 5 days and tumors were weighed after sacrifice (*n* = 6 for T47D sh-control; *n* = 5 for T47D sh-C1GALT1). **(C)** C1GALT1 expression level is down-regulated in sh-C1GALT1 xenograft tumor tissues confirmed by immunohistochemistry and Western blotting. Increased VVA binding was detected by Western blotting in C1GALT1 knockdown T47D tumors. Decreased Ki-67 staining was observed in C1GALT1 knockdown T47D tumors. Scale bar = 50 μm. **(D)** Stable overexpression of C1GALT1 in MCF-7 cells with pcDNA3.1/C1GALT1 plasmid was confirmed by Western blotting. **(E)** Overexpression of C1GALT1 in MCF-7 cells enhanced tumor weight and tumor growth *in vivo* (*n* = 6 for MCF-7 Mock; *n* = 5 for MCF-7 C1GALT1). **(F)** C1GALT1 expression level is up-regulated in C1GALT1 overexpressing tumors confirmed by immunohistochemistry and Western blotting. Decreased VVA binding was detected by Western blotting in C1GALT1 overexpressing MCF-7 tumors. Increased Ki-67 staining was observed in C1GALT1 overexpressing MCF-7 tumors. *p < 0.05; **p < 0.01.

To investigate the effects of C1GALT1 on tumor growth *in vivo*, stable transfectants of C1GALT1 knockdown T47D cells and C1GALT1 overexpressing MCF-7 cells were xenografted in NOD/SCID mice. Knockdown of C1GALT1 in T47D cells and overexpression of C1GALT1 in MCF-7 cells were confirmed by Western blotting (Figure 4A and 4D). Our results show that knockdown of C1GALT1 suppressed tumor growth and tumor weight in T47D cells compared with control (Figure 4B). In contrast, overexpression of C1GALT1 promoted MCF-7 tumor growth and tumor weight compared with mock (Figure 4E). C1GALT1 knockdown and overexpression in tumor xenografts were confirmed by immunohistochemistry and Western blotting (Figure 4C and 4F). Immunohistochemistry shows decreased Ki-67 staining in C1GALT1 knockdown T47D tumors (Figure 4C), but increased Ki-67 staining in C1GALT1 overexpressing MCF-7 tumors (Figure 4F). Furthermore, the glycan changes in tumor xenografts were tested by VVA blotting (Figure 4C and 4F). Western blots show increased VVA binding in C1GALT1 knockdown T47D tumors (Figure 4C) and decreased VVA binding in C1GALT1 overexpressing MCF-7 tumors (Figure 4F). Consistent with *in vitro* cell growth analyses, these results suggest that C1GALT1 expression can promote tumor growth *in vivo*.

### C1GALT1 regulates O-glycosylation of proteins in breast cancer cells

**Figure 5:**
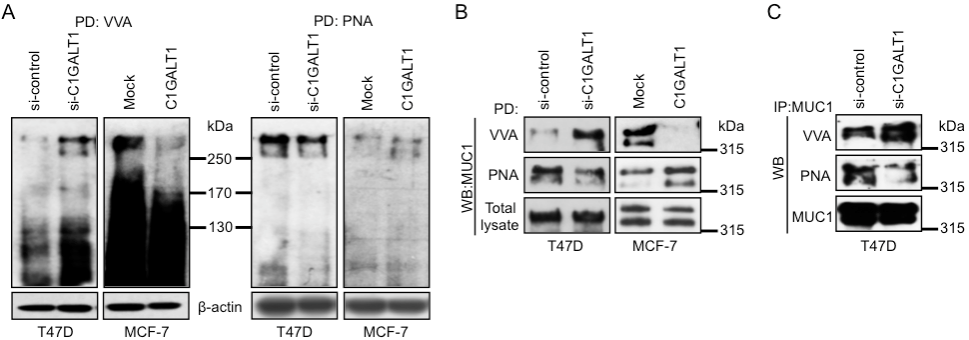
C1GALT1 regulates O-glycosylation of proteins in breast cancer cells **(A)** Effects of C1GALT1 on O-glycans of cell surface proteins. Cell surface proteins were surface biotinylated and then pulled down with VVA or PNA lectin beads for 16 hours. **(B)** Effects of C1GALT1 on O-glycans of MUC1-N analyzed by VVA pull down assay. Knockdown of C1GALT1 in T47D cells increased VVA binding to MUC1-N, while overexpression of C1GALT1 in MCF-7 cells decreased VVA binding to MUC1-N. MUC1-N was detected by M2C5 antibody. Total MUC1-N was used as loading control. **(C)** Western blotting of immunoprecipitated MUC1-N with VVA lectin. In immunoprecipitation assay, lysates with 300 μg proteins were incubated with M2C5 antibody or IgG beads for 16 hours, and biotinylated VVA lectin was used to detect the carbohydrate changes on MUC1-N. Total MUC1-N was used as control.

Next, we investigated whether C1GALT1 affects O-glycosylation in breast cancer cells. C1GALT1 knockdown T47D and C1GALT1 overexpressing MCF-7 cells were surface biotinylated and lysates were incubated with VVA lectin beads or peanut agglutinin (PNA) lectin beads for lecin pull-down assay. PNA is used to detect core 1 structure. Immunoblotting results reveal that knockdown of C1GALT1 enhanced VVA binding and decreased PNA binding to cell surface proteins with molecular weight above 250 kDa in T47D cells and MCF-7 cells (Figure 5A). These findings indicate that C1GALT1 modifies O-glycosylation of cell surface proteins in breast cancer cells.

### C1GALT1 regulates O-glycan structures on MUC1

MUC1 is a highly O-glycosylated transmembrane protein that is frequently overexpressed and aberrantly glycosylated in various epithelial cancers, particularly in breast cancer [24, 25] and plays important roles in breast cancer formation. The presence of core 1 structure on MUC1 has been reported to associate with its recycling [26] and protects breast cancer cells from immune surveillance [27]. Since MUC1 is a high molecular weight (>250 kDa) O-glycoprotein, we postulate that MUC1 is a possible acceptor substrate of C1GALT1. We therefore hypothesize that C1GALT1 can modify MUC1 O-glycosylation, affect MUC1 signaling, and in turn regulate breast cancer cell behaviors. To investigate whether O-glycans on MUC1 can be modified by C1GALT1, we performed VVA and PNA lectin pull-down assays. Knockdown of C1GALT1 increased VVA binding but decreased PNA binding to MUC1-N in T47D cells (Figure 5B, left). Similar results were also observed in MDA-MB-231 and SKBR3 cells (Supplementary Figure S4). Conversely, overexpression of C1GALT1 decreased VVA binding but increased PNA binding to MUC1-N in MCF-7 cells (Figure 5B, right). To further confirm these findings, immunoprecipitation (IP) of MUC1-N followed by Western blotting (WB) with VVA or PNA lectins shows enhanced VVA binding but decreased PNA binding to MUC1-N in C1GALT1 knockdown T47D cells (Figure 5C). These results suggest that C1GALT1 can modify O-glycan structures on MUC1 in breast cancer cells.

### C1GALT1 regulates MUC1-N shedding into conditioned medium and MUC1-C translocation into the nucleus

**Figure 6:**
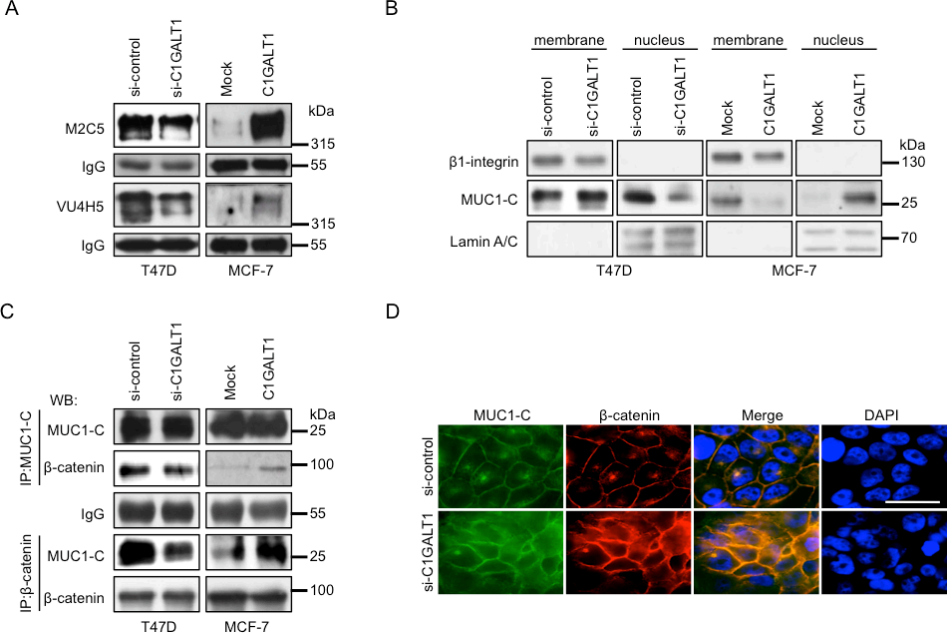
C1GALT1 regulates MUC1-N shedding and MUC1-C distribution in breast cancer cells **(A)** Effects of C1GALT1 on MUC1-N shedding. Knockdown of C1GALT1 in T47D cells with siRNA (si-C1GALT1) decreased MUC1-N shedding into the conditioned medium compared with si-control, while overexpression of C1GALT1 in MCF-7 cells increased MUC1-N shedding into the conditioned medium compared with mock. The conditioned medium was collected after 3 days of incubation and anti-MUC1-N antibodies (M2C5 and VU4H5 clones) were used for immunoprecipitation. Mouse IgG was used as antibody loading control. **(B)** Effects of C1GALT1 on nuclear translocation of MUC1-C. Knockdown of C1GALT1 in T47D cells suppressed MUC1-C accumulation in the nucleus, while overexpression of C1GALT1 in MCF-7 cells enhanced MUC1-C accumulation in nucleus. LaminA/C was used as internal control for the nuclear fraction; β1-integrin was used as control for membrane fraction. Cell fractionation was conducted and protein levels were analyzed by Western blotting. **(C)** The interaction of MUC1-C with β-catenin in T47D and MCF-7 cells. Co-immunoprecipitation assays show that knockdown of C1GALT1 in T47D cells decreased MUC1-C association with β-catenin. In contrast, overexpression of C1GALT1 in MCF-7 cells increased MUC1-C association with β-catenin. **(D)** Effects of C1GALT1 on sub-cellular localization of MUC1-C and β-catenin. Immunofluorescence staining results show that knockdown of C1GALT1 in T47D cells decreased MUC1-C (green) accumulation in nucleus and associated with β-catenin (red). DAPI was used to stain nucleus. Scale bars = 10 μm.

MUC1-C is a transmembrane subunit of MUC1 glycoprotein that accumulates in the cytoplasm and is associated with β-catenin to translocate into the nucleus in triggering expression of cancer-related oncogenes associated with tumor progression [28, 29]. To determine the mechanisms by which C1GALT1 regulates breast cancer cell malignant phenotypes, M2C5 and VU4H5 clones of MUC1 antibodies were used to analyze MUC1 shedding and signaling. Immunoblotting results reveal that knockdown of C1GALT1 in T47D cells show decreased MUC1-N shedding into conditioned medium (Figure 6A). In contrast, overexpression of C1GALT1 in MCF-7 cells increased MUC1-N shedding into conditioned medium. These findings suggest that shedding of MUC1-N is regulated by expression of C1GALT1.

Next, cell fractionation assay was performed to analyze the effects of C1GALT1 expression on MUC1-C distribution. The results show that knockdown of C1GALT1 suppressed MUC1-C translocation into the nucleus and caused slight increase in the membrane fraction of MUC1-C in C1GALT1 knockdown T47D cells compared with control (Figure 6B). In contrast, overexpression of C1GALT1 in MCF-7 cells increased MUC1-C translocation into the nucleus and decreased MUC1-C in the membrane fraction compared with mock (Figure 6B). These results suggest that C1GALT1 regulates MUC1-C translocation into the nucleus.

### C1GALT1 regulates MUC1-C/β-catenin signaling pathway

To analyze the effect of C1GALT1 expression on interaction of MUC1-C with β-catenin, co-immunoprecipitation assay was performed. Knockdown of C1GALT1 in T47D cells decreased MUC1-C association with β-catenin compared with control (Figure 6C). In contrast, overexpression of C1GALT1 in MCF-7 cells increased MUC1-C association with β-catenin compared with mock (Figure 6C). Consistently, immunofluorescence staining shows that knockdown of C1GALT1 suppressed MUC1-C translocation into the nucleus and co-localization with β-catenin in T47D cells (Figure 6D). ERK phosphorylation has previously been reported as a downstream signaling pathway of MUC1-C in breast cancer cells [29, 30]. Our results show that knockdown of C1GALT1 decreased p-ERK (phospho-ERK) levels in T47D cells, whereas overexpression of C1GALT1 increased p-ERK levels in MCF-7 cells (Supplementary Figure S5). These findings suggest that C1GALT1 affects MUC1-C/β-catenin signaling pathway in breast cancer cells.

### C1GALT1 promotes cell growth through MUC1-C pathway

**Figure 7:**
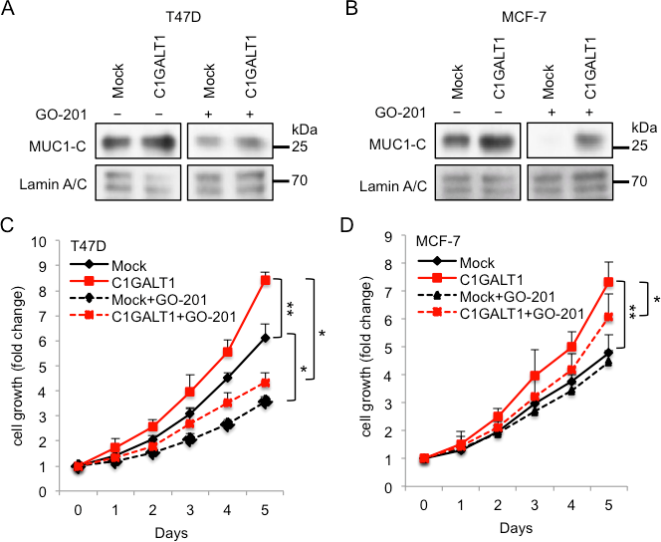
C1GALT1 promotes cell growth through MUC1-C pathway GO-201 suppressed C1GALT1-inceased MUC1-C accumulation in nucleus in T47D **(A)** and MCF-7 cells **(B)**. LaminA/C was used as internal control of nuclear fraction. GO-201 was added to culture medium and incubated for 3 days. **(C)** MTT assay shows that GO-201 suppressed C1GALT1-mediated cell growth in T47D cells. GO-201 was added to culture medium and incubated for 5 days. **(D)** GO-201 suppressed C1GALT1-mediated cell growth in MCF-7 cells. *p < 0.05; **p < 0.01.

To double confirm the molecular mechanisms of C1GALT1 in regulating MUC1-C translocation into the nucleus and the breast cancer cell growth, GO-201, peptide that binds to MUC1-C CQC motif which inhibits MUC1 signaling pathway [29] was used. Stable clones of C1GALT1 overexpressing T47D cells were established and confirmed by Western blotting (data not shown). After treatment with GO-201 for 3 days, the C1GALT1-enhanced accumulation of MUC1-C in nucleus was decreased in both T47D (Figure 7A) and MCF-7 cells (Figure 7B). Furthermore, MTT cell survival analysis reveals that GO-201 significantly suppressed C1GALT1-enhanced cell viability in T47D (Figure 7C) and MCF-7 cells (Figure 7D). These findings suggest that C1GALT1 affects breast cancer cell growth through MUC1-C signaling pathway.

## DISCUSSION

Mucin-type O-glycosylation is fundamentally required for the normal functions of mammalian cells. Dysregulated expression of mucin-type O-glycans has evidently lead to aberrant cellular response, particularly in cancer cells [4, 31]. We recently reported that C1GALT1 is up-regulated in colorectal cancer [16] and hepatocellular carcinoma [15]. Up-regulation of C1GALT1 enhanced malignant phenotypes in colorectal cancer and hepatocellular carcinoma through FGFR2 and MET signaling pathways, respectively. Here we report that C1GALT1 mRNA and protein are frequently overexpressed in breast tumor tissues and high C1GALT1 expression correlates with higher histological grade and tumor stage. *In vitro* and *in vivo* results show that up-regulation of C1GALT1 promotes breast cancer cell growth. The mechanistic investigation indicates that C1GALT1 regulates the O-glycan structures on MUC1 oncoprotein, and promotes MUC1-N shedding and MUC1-C/β-catenin signaling pathway in breast cancer cells. This study further supports the critical role of O-glycosylation in breast cancer development.

MUC1 consists of a heavily O-glycolsylated N-terminal subunit, MUC1-N, and a C-terminal transmembrane subunit, MUC1-C, which are non-covalently linked [28, 32]. MUC1 is expressed on the apical surface of normal secretory epithelial cells to form barriers against pathogens and maintains an apical-basal polarity of the epithelial cell [32]. Overexpression of MUC1 is a common feature of breast cancer [33]. In this study, we found that overexpression of C1GALT1 decreases Tn antigens and increases T antigens on cell surfaces and MUC1 and enhances breast cancer cell malignant behaviors. However, previous studies show that increased Tn and sTn antigens correlate with poor prognosis in various cancers [9, 34]. The explanation for this discrepancy may be that malignant phenotypes are regulated by many factors and not all of them can increase Tn expression. Moreover, the effect of Tn antigens on cancers may be influenced by their different genetic backgrounds. It should be noted here that the correlation between Tn expression and breast cancer prognosis is still unclear.

Increasing evidence reveals that the extracellular subunit of MUC1 is shed from cancer cell membrane and released into the blood stream [36–38]; and aberrant expression of T antigen regulates MUC1 trafficking and recycling [26]. Interestingly, we found that C1GALT1 knockdown decreases MUC1-N shedding, whereas C1GALT1 overexpression enhances MUC1-N shedding in breast cancer cells. It has recently been reported that O-glycosylation of Notch by Galnt11 enhances shedding of Notch by ADAM17 and activates signaling by increased NICD [39]. Therefore, it is likely that conformational changes in MUC1 caused by C1GALT1 modification instead of steric hindrance of complex O-glycans play the predominant role in the proteolytic cleavage of MUC1 in breast cancer cells. However, further study is required to address the detailed mechanisms by which C1GALT1 affects the proteolysis of MUC1. Since T antigens can be further extended to form more complex O-glycans, it should be noted that the effects of C1GALT1 on MUC1 and cancer cell behaviors could be resulted from core 1 and core 1 derived O-glycans.

MUC1-C, consisting of a 58 amino acids extracellular domain, a 28 amino acids transmembrane domain, and a 72 amino acids cytoplasmic domain, is involved in many cellular biological functions by effecting dimerization and association with β-catenin to translocation into the nucleus to turn on cancer-related genes expression [29, 40]. Furthermore, galectin-3 interacts with T antigens on MUC1 to promote cancer metastasis [23]. Here, we demonstrate that C1GALT1 expression regulates O-glycans on MUC1-N and promotes MUC1-C association with β-catenin and nuclear translocation in breast cancer cells. These findings indicate that up-regulation of C1GALT1 promotes breast tumor growth through modified glyco-phenotypes of MUC1-N and MUC1-C signaling pathway. An inhibitory small peptide GO-201 specifically targeting the CQC residues of MUC1-C cytoplasmic domain has been well established to prevent MUC1-C dimerization and activation in breast cancer development [29, 41]. Indeed, we found that GO-201 significantly suppressed C1GALT1-mediated cell viability and inhibited MUC1-C translocation into the nucleus in T47D and MCF-7 cells. This finding further confirms that C1GALT1 regulates breast cancer cell growth through MUC1-C signaling pathway.

Although many therapeutic antibodies and diagnostic tools are present, however, improvement on the efficacy and specificity of these therapeutic agents in the management of breast cancer is still required [42, 43]. For many years, researches have been focusing on specific glycoforms of MUC1 as diagnostic and therapeutic targets [44, 45]. Here, we report that C1GALT1 is a critical regulator for O-glycan structures on MUC1-N and C1GALT1 promotes breast cancer progression through MUC1 shedding and MUC1-C/β-catenin signaling pathways. This study provides a new insight into the function of C1GALT1 in regulating O-glycosylation and malignant phenotypes of breast cancer and suggests C1GALT1 as a target of therapeutic drug development.

## MATERIALS AND METHODS

### Immunohistochemistry

Breast cancer tissue microarray (BRC1021) was obtained from Pantomics Inc. for immunohistochemical staining. Monoclonal anti-C1GALT1 antibody (Santa cruz) was used and detected with Super Sensitive Link-Label immunohistochemistry Detection System (BioGenex). The specific staining was visualized with 3,3-diaminobenzidine liquid substrate system (Sigma) and counterstained with hematoxylin (Sigma).

### Cell lines and cell culture

MCF-7 and MDA-MB-231 cells were kindly provided by Dr. Ming-Shyue Lee (National Taiwan University, Taiwan), and MDA-MCF-10A, MDA-MB-435, SKBR3 and T47D cells were obtained from Dr. Tang-Long Shen (National Taiwan University, Taiwan). Inhibitory small peptide, GO-201 (Sigma), was applied to the culture medium at a final concentration of 2.5 μM and incubated for 3 days. Breast cancer cells were cultured in Dulbecco's modified Eagle's medium (DMEM; Invitrogen) containing 10% FBS (Invitrogen), 100 IU/mL penicillin, and 100 μg/mL streptomycin (Invitrogen) in a humidified tissue culture incubator at 37°C and 5% CO_2_ atmosphere. All cell lines were authenticated by the provider on the basis of morphology, antigen expression, growth, DNA profile, and cytogenetics.

### Transfection

C1GALT1/pcDNA3.1 was constructed as reported previously [15] for overexpression of C1GALT1 in MCF-7 and T47D cells with Lipofectamine 2000 (Invitrogen). pcDNA3.1/myc-His (Invitrogen) was used as control. Stable pooled clones were selected and maintained with 400 μg/mL G418. In transient knockdown experiments, C1GALT1 specific siRNA and control siRNA (Invitrogen) were used to transfect SKBR3, T47D and MDA-MB-231 cells with Lipofectamine RNAiMAX (Invitrogen) for 2 days. For stable knockdown of C1GALT1 in T47D cells, sh-C1GALT1/pLKO.1-puro and sh-Ctrl/pLKO.1-puro (RNAi Core, Academia Sinica) were used in lentivirus-based infection system and stable pooled clones were selected and maintained with 1 μg/mL puromycin (Sigma).

### MTT assay

Cells (2 × 10^3^) in 100 μl complete DMEM were seeded in 96-well plates. Ten microliters of 5 mg/ml 3-(4,5-dimethyl-2-thiazolyl)-2,5-diphenyl-2H-tetrazolium bromide solution (MTT; Sigma) was added to each well for the indicated times and incubated at 37°C for 4 hours, after which 100 μl 10% SDS in 0.01N HCl was added to dissolve the MTT formazan crystals. The resultant optical density was measured spectrophotometrically at dual wavelengths, 550 and 630 nm.

### *In vivo* xenograft tumor growth model

For *in vivo* xenograft tumor growth experiment, 5 × 10^6^ of T47D and MCF-7 transfectants were subcutaneously injected into 6-week-old female NOD/SCID mice (National Laboratory Animal Center, Taiwan). Tumor size was measured every 5 days. The mice were sacrificed at day 40 and tumors were excised for further experiments. The tumors were weighed and fixed with paraformaldehyde for immunohistochemistry staining or lysed with NP-40 lysis buffer for Western blot. Animal experiments were reviewed and approved by the Institutional Animal Care and Use Committee IACUC) of College of Medicine, National Taiwan University.

### Flow cytometry

Cells (1 × 10^5^) were resuspended in 100 μl PBS with 0.5% BSA, pretreated with neuraminidase (Sigma) at 37°C for 30 minutes, and then incubated with VVA lectin-FITC (Vector Laboratories) at 1:100 in PBS with 0.5% BSA on ice for 30 minutes. After washing twice, the fluorescence intensity of 1 × 10^4^ cells per sample in 100 μl PBS was analyzed by flow cytometry (FACS Calibur; BD Pharmingen). Samples without lectin binding serve as negative controls.

### Western blot analysis and lectin pull-down assay

Antibodies against C1GALT1, MUC1 (clones M2C5 and VU4H5) (Santa Cruz), MUC1-C (Thermo Scientific), LaminA/C (Cell Signaling) and β-actin (Sigma) were used for Western blotting. In surface biotinylation assay, 1 × 10^6^ cells were incubated with Sulfo-NHS-SS-Biotin (Thermo Scientific) at room temperature for 30 minutes and cell lysate were purified with NP-40 lysis buffer by centrifuge for l5 minutes at 4°C. To analyze the modifications of glycoproteins, 300 μg total cell lysates were prepared and incubated with VVA or PNA agarose beads (Vector Laboratories) at 4°C overnight. After washing twice, pulled-down proteins were subjected to Western blot analysis. In immunoprecipitation assays, antibodies were coupled to protein G sepharose beads (GE Heathcare). Horseradish peroxidase (HRP)-conjugated mouse, rabbit (Jackson ImmunoResearch Laboratories) and streptavidin (BD Biosciences) secondary antibodies were used in Western blotting.

### Immunofluorescence microscopy

T47D cells were seeded on 10-mm cover glasses and transfected with siRNA as described above. After washing twice, cells were fixed with 4% paraformaldehyde for 15 minutes and permeabilized with 0.1% Triton X-100 and incubated with the MUC1-C (Thermo Scientific) and β-catenin (BD Biosciences) antibodies followed by anti-hamster IgG-FITC and anti-mouse IgG-Texas Red antibodies. The images were captured by Zeiss microscope system.

### Quantitative (Q) RT-PCR analysis

Total RNA was purified by Trizol reagent (Invitrogen) and 1 μg RNAs were prepared for cDNA synthesis by iScript™ cDNA synthesis kit (Bio-Rad). *C1GALT1* and *GAPDH* Q-PCR primer sequences were designed as previously described [15]. The relative quantity of gene expression was analyzed by Quantitative PCR System Mx3000P (Stratagene) and normalized to *GAPDH* by MxPro software (Stratagene).

### Cell fractionation

Cell fractionation assay was performed as described [46]. Cells were resuspended with digitonin extraction buffer and centrifuged at 500 g for 10 minutes followed by Triton X-100 extraction and centrifuged at 5000 g for 10 minutes to obtain supernatant as the membrane fraction. Nuclear fraction was extracted with Tween/DOC extraction buffer and centrifuged at 7000 g for 10 minutes.

### Statistical analyses

Results are presented as the mean ± SD and Student *t*-test was used to compare differences between two experimental groups. All statistical analyses are 2-sided. C1GALT1 scoring intensity in correlation with histological grade and tumor stage was analyzed by Spearmen Rank Correlation and Chi-square analysis. *p* < 0.05 is considered statistically significant.

## SUPPLEMENTARY MATERIALS AND METHODS


