# Supplementary material for: Up-regulation of C1GALT1 promotes breast cancer cell growth through MUC1-C signaling pathway
Source: Oncotarget. 2015 Jan 27;6(8):6123–35. doi: 10.18632/oncotarget.3045 (PMC4467426; doi:10.18632/oncotarget.3045)
Supplement: Supplementary file 1 [file oncotarget-06-6123-s001.pdf]

SUPPLEMENTARY MATERIALS AND METHODS

T-synthase activity assay

Fluorescence-base T-synthase activity assay was conducted as described [1]. GalNAc-α-(4-MU) and UDP-Gal were purchased from Sigma-Aldrich. *O*-Glycosidase (40,000,000 units/mL) was obtained from New England Biolabs. The cell extracts were purified as the previous report [2] and standardized protein concentration by Bradford protein assay (Bio-Rad). Ninety-six well black microtiter plate with flat bottom (Perkin Elmer) was used for the assay. The reaction containing 500 μM GalNAc-α-(4-MU), 500 μM UDP-Gal, 20 mM $MnCl_2$, 0.2% Triton X-100, and 800 units of *O*-glycosidase in 50 mM MES-NaOH buffer (pH 6.8), and 10 μl cell extract to 50 μl of total reaction volume. The blank reaction was prepared by replaced the UDP-Gal with H_2O in the 50 μl reaction volume. The reactions were incubated at 37°C for 90 minutes. Then, 100 μl of 1.0 M glycine-NaOH (pH 10.0) were added to each well to stop reaction. The relative fluorescence intensity was measured by Beckman Coulter PARADIGM (Ex: 355nm and Em: 460 nm). The results of the fold change of activity of T-synthase were calculated as described in a previous report [2].

REFERENCES

1. Ju T, Xia B, Aryal RP, Wang W, Wang Y, Ding X, Mi R, He M, Cummings RD. A novel fluorescent assay for T-synthase activity. Glycobiology. 2011; 21:352–362.
2. Ju T, Cummings RD. A fluorescence-based assay for Core 1 beta3galactosyltransferase (T-synthase) activity. Methods in molecular biology (Clifton, NJ). 2013; 1022:15–28.

SUPPLEMENTARY FIGURES

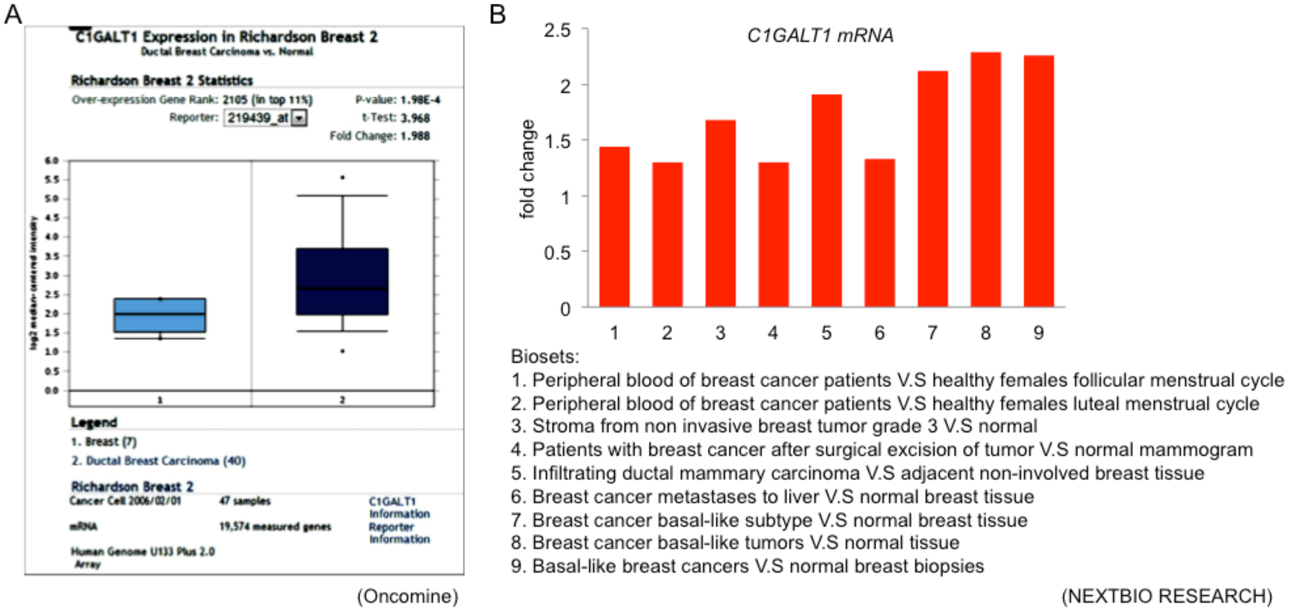

Supplementary Figure S1: *C1GALT1* mRNA is frequently up-regulated in ductal carcinoma and basal-like breast cancer. (A) Oncomine database (Richardson Breast 2) shows that ***C1GALT1*** mRNA is up-regulated in ductal breast carcinoma (***n*** = 40) compared with normal breast tissues (***n*** = 7). Fold change 1.988, ***p*** < 0.0004. **(B) *C1GALT1*** mRNA is overexpressed in breast cancer across selected breast cancer biosets. 1. Peripheral blood of breast cancer patients V.S healthy females follicular menstrual cycle (Fold Change 1.44). 2. Peripheral blood of breast cancer patients V.S healthy females luteal menstrual cycle (Fold Change 1.3). 3. Stroma from non-invasive breast tumor grade 3 V.S normal (Fold Change 1.68). 4. Patients with breast cancer after surgical excision of tumor V.S normal mammogram (Fold Change 1.3). 5. Infiltrating ductal mammary carcinoma V.S adjacent non-involved breast tissue (Fold Change 1.91). 6. Breast cancer metastases to liver V.S normal breast tissue (Fold Change 1.33). 7. Breast cancer basal-like subtype V.S normal breast tissue (Fold Change 2.12). 8. Breast cancer basal-like tumors V.S normal tissue (Fold Change 2.29). 9. Basal-like breast cancers V.S normal breast biopsies (Fold Change 2.26).

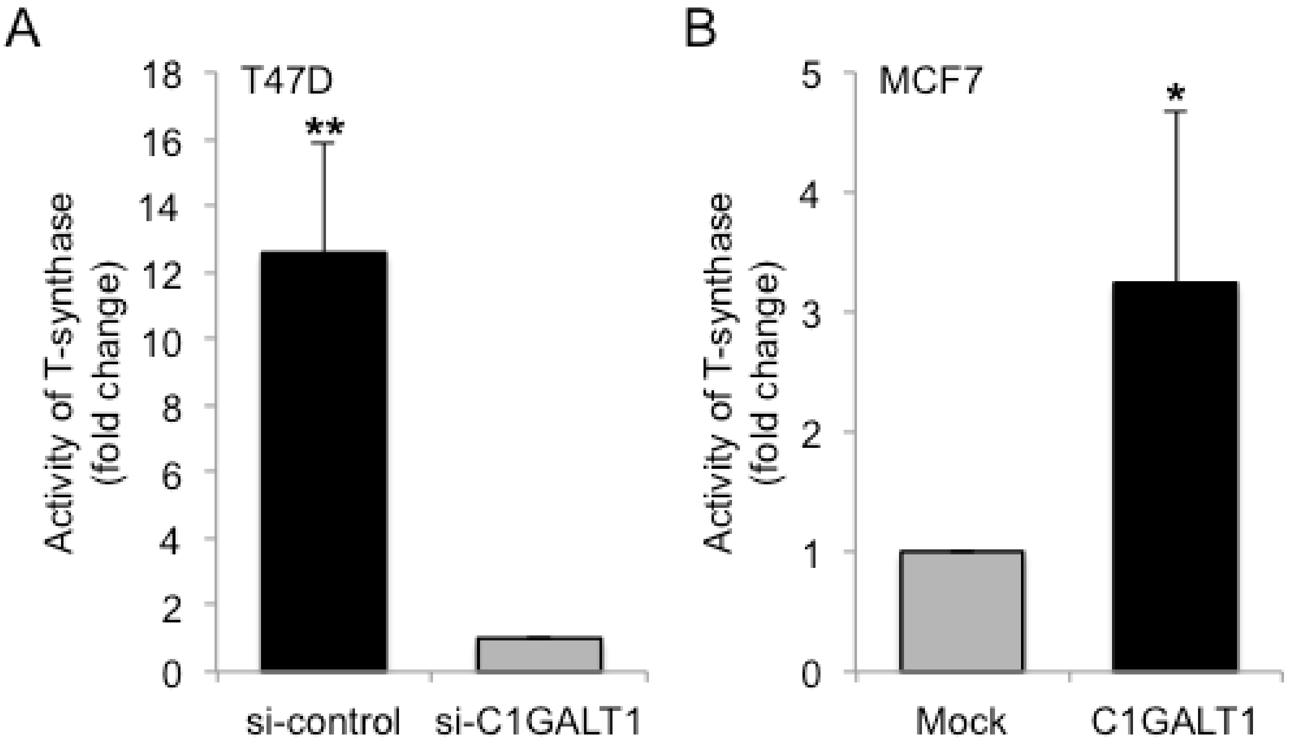

Supplementary Figure S2: C1GALT1 regulates T-synthase activity in T47D and MCF-7 cells. **(A)** Knockdown of C1GALT1 in T47D cells decreased T-synthase activity compared with si-control. **(B)** Overexpression of C1GALT1 increased T synthase activity compared with mock. The cell extracts were used for T-synthase activity assay. The results obtained were normalized with si-control or mock and are presented as fold change. *$p < 0.05$; **$p < 0.01$.

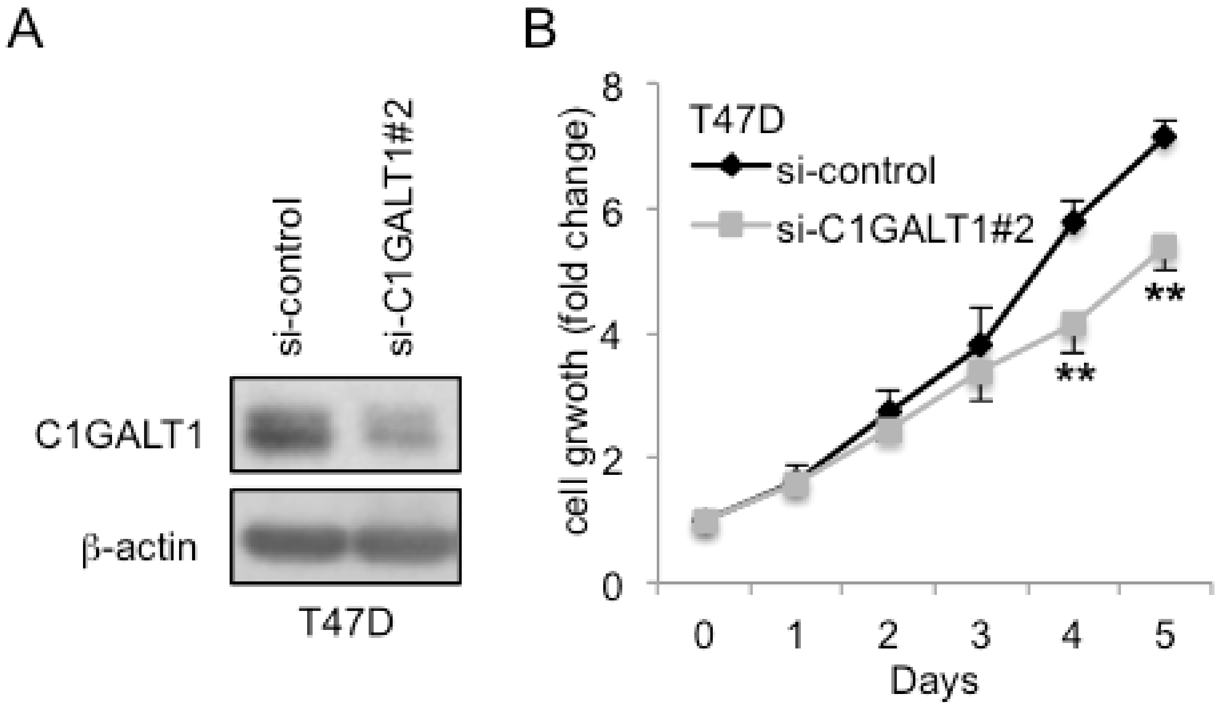

Supplementary Figure S3: Knockdown of C1GALT1 by si-C1GALT1#2 suppresses T47D cell growth *in vitro*. (A) Knockdown of C1GALT1 by si-C1GALT1#2 was confirmed by Western blotting in T47D cells. **(B)** Knockdown of C1GALT1 by si-C1GALT1#2 in T47D cells suppressed cell viability analyzed by MTT assay. *$p < 0.05$; **$p < 0.01$.

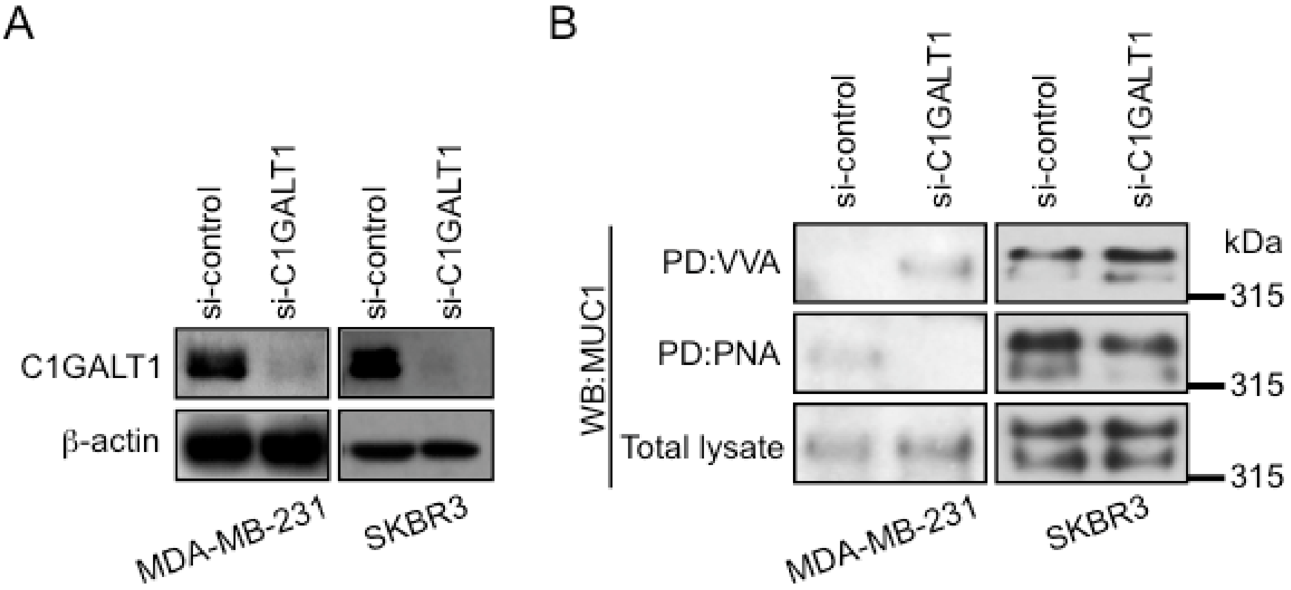

Supplementary Figure S4: Knockdown of C1GALT1 modifies O-glycans on MUC1-N in MDA-MB-231 and SKBR3 breast cancer cells. (A) C1GALT1 was knocked down in MDA-MB-231 and SKBR3 breast cancer cells. C1GALT1 specific siRNA (si-C1GALT1) was used for C1GALT1 knockdown in breast cancer cells and non-targeting siRNA (si-control) was used as control. **(B)** Knockdown of C1GALT1 in MDA-MB-231 and SKBR3 cells increased VVA binding but decreased PNA binding to MUC1-N in lectin pull-down assay. MUC1-N was detected by M2C5 monoclonal antibody. Total MUC1-N in whole cell lysate was used as control.

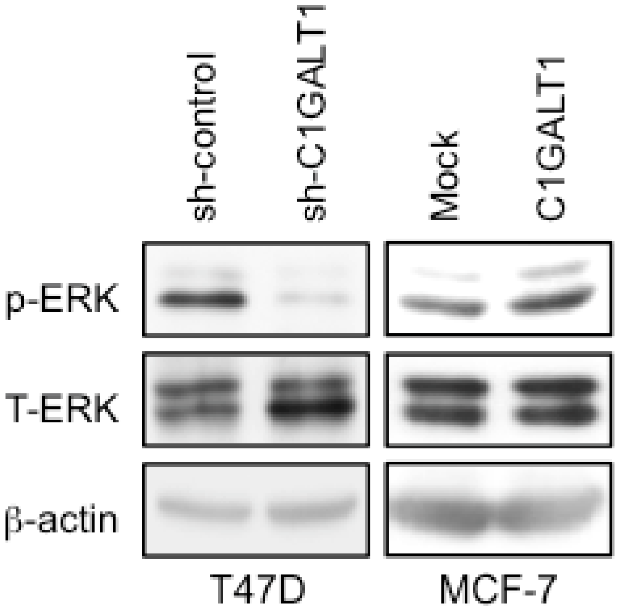

Supplementary Figure S5: C1GALT1 regulates ERK phosphorylation in T47D and MCF-7 cells. Phosphorylation of ERK was analyzed by Western blotting. C1GALT1 knockdown in T47D cells decreased ERK phosphorylation. Overexpression of C1GALT1 in MCF-7 cells increased ERK phosphorylation. Total ERK and β-actin were detected as internal controls.
